# Effect of *Curcuma longa* extract on reproduction function in mice and testosterone production in Leydig cells

**DOI:** 10.1111/jcmm.18303

**Published:** 2024-04-13

**Authors:** Yisheng Zhang, Liu Yang, Shan Xue, Yichang Zhang, Zihan Li, Min Zhang, Guoyin Kai, Juan Li

**Affiliations:** ^1^ Department of Pharmacy Wuhan Hospital of Traditional Chinese Medicine Wuhan Hubei China; ^2^ Hubei Province Key Laboratory of Traditional Chinese Medicine Resource and Chemistry, Hubei Shizhen Laboratory, Hubei University of Chinese Medicine Wuhan Hubei China; ^3^ College of pharmacy Zhejiang Chinese Medical University Hangzhou Zhejiang China

**Keywords:** *Curcuma longa* extract, kidney‐Yang deficiency, Leydig cells, steroidogenic enzymes, testosterone production

## Abstract

*Curcuma longa,* best known for its culinary application as the main constituent of curry powder, has shown potential impact on the reproductive system. This study aimed to investigate the efficacy of *Curcuma longa* extract (CLE) on Kidney‐Yang deficiency mice induced by hydrocortisone and the possible roles in testosterone secretion in Leydig cells. We evaluated male sexual behaviour, reproductive organ weight, testosterone levels, and histological tissue changes in hydrocortisone‐induced mice. CLE effectively reversed hydrocortisone‐induced Kidney‐Yang deficiency syndrome by improving sexual behaviour, testis and epididymis weight, testosterone levels and reducing pathological damage. Our in vitro study further indicated that CLE stimulated testosterone production via upregulating the mRNA and protein expression of steroidogenic enzymes in Leydig cells. It significantly improved H89‐inhibited protein expression of StAR and cAMP‐response element‐binding (CREB), as well as melatonin‐suppressed StAR protein expression. The data obtained from this study suggest that CLE could alleviate Kidney‐Yang deficiency symptoms and stimulate testosterone production by upregulating the steroidogenic pathway. This research identifies CLE as a potential nutraceutical option for addressing testosterone deficiency diseases.

## 1 INTRODUCTION

*Curcuma longa* L., commonly known as turmeric, is a perennial rhizomatous herb from Zingiberaceae that widely grows in southeast Asia, China, India and Pakistan. The United States Food and Drug Administration classifies turmeric as nutraceutical food that is generally recognized as safe. It is best known for its culinary application as the main constituent of curry powder and is extensively used as a spice, food preservative and natural dye in the food, pharmaceutical and cosmetics industries.^1^ Traditional Indian medicine uses *C. longa* as an aromatic stimulant and carminative, while traditional Chinese medicine uses it to promote blood circulation and pain relief.^2^, ^3^ It is also well known for treating conditions ranging from rheumatoid arthritis and diabetes to Alzheimer's disease and cancer.^4^ Polyphenolic curcuminoids, consisting of curcumin desmethoxycurcumin and bisdemethoxycurcumin are the critical components of turmeric.^5^ Curcuminoids have multiple beneficial properties, such as anti‐inflammatory, antioxidant, chemoprophylaxis and chemotherapeutic activities.^6^, ^7^

Various data are available concerning the impact of *C. longa* on male reproductive systems. In vivo studies implicate that aqueous rhizome extracts have reversible antifertility and suppress spermatogenesis effects on male mice,^8^ and methanol extract may be useful in the management of benign prostatic hyperplasia in male rats.^9^ They arrested spermatogenesis and affected the androgen synthesis by inhibiting the Leydig cell function or the hypothalamus‐pituitary axis (HPA).^10^ Simultaneously, several studies emphasize the involvement of turmeric and curcumin in improving sexual function and sex hormones profiling.^11^, ^12^ Furthermore, it has been shown to alleviate adverse reproductive outcomes of various physiological or environmental factors and exerts advantageous effects on the sexual glands, testis, and ovary via potent antioxidant, anti‐inflammatory and membrane‐stabilizing properties.^13^, ^14^, ^15^ Thus, different effects of turmeric might depend on the doses, and various models studied.

Kidney‐Yang Deficiency syndrome is closely related to multiple disordered metabolic pathways, endocrine, and reproductive systems.^16^ The functional disorder, to some extent, of the HPA, is the crucial mechanism for forming ‘Kidney‐Yang deficiency syndrome’.^17^ Hydrocortisone administration resulted in diminished steroid hormone production from the adrenal cortex, thereby disrupting metabolic pathways as well as the endocrine and reproductive systems.^18^ Meanwhile, testicular Leydig cells regulate the development and activity of the male reproductive tract and external sex characteristics and maintenance spermatogenesis is a crucial contributor to androgen synthesis and secretion. Therefore, the present in vitro and in vivo study aimed to investigate the efficacy of CLE on hydrocortisone‐induced Kidney‐Yang deficiency mice and the possible roles in testosterone secretion in Leydig cells.

## 2 MATERIALS AND METHODS

### 2.1 Primary reagents

Collagenase, DMEM/F‐12 medium, horse serum, and fetal bovine serum were from GIBCO (California, USA). MTT and DMSO were from Sigma (St.Louis, USA). Testosterone Assay Kit was purchased from NanjingJiancheng Institute of Biological Engineering (Nanjing, China). FastQuant Reverse transcription kit and TIANGEN SuperReal PreMix Plus kits (SYBR Green) were purchased from Tiangen Biotech (Beijing, China). Pierce ECL Western Blotting Substrate was from Thermo Scientific. Anti–GAPDH Rabbit pAb (GB11002) and HRP‐conjugated Goat Anti‐Rabbit IgG (GB23303) were from Servicebio (Wuhan, China). StAR Polyclonal Antibody (A16432), HSD3B1 Rabbit pAb (A8035), NR5A1 Polyclonal Antibody (A1657), and CREB (A11989) were from ABclonal (Wuhan, China). H89 were from MedChemExpress (New Jersey, USA). Human chorionic gonadotropin (hCG) was from National Institutes for Food and Drug Control (Beijing, China). JKSQP (Jinkui Shenqi Pill) was from Beijing Tongrentang Company.

### 2.2 Sample preparation and high‐performance liquid chromatography (HPLC) analysis

Rhizoma of *C. longa* was collected from Hubei Tianji Chinese Medicine Pieces Co., Ltd. in China and identified by *Prof*. Keli Chen. The voucher specimen (NO.170908) for future references has been kept in our laboratory. The dried rhizome of *C. longa* was successively extracted with petroleum ether and 80% ethanol for 2 h by refluxing three times. After the extract was filtered, the decoction was concentrated on a vacuum evaporator to yield 30% of the total yield.

HPLC analysis was performed on a Dionex HPLC system with a P680 Pump, an Agilent ZORBAX SB‐Aq C18 column (5.6 mm × 250 mm, 5 μm) and a UVD 170 U variable wavelength UV–Vis detector. Data were collected and processed using ‘Chromeleon version 6.0’ software. The mobile phase consisted of acetonitrile: 4% glacial acetic aqueous acid solution (48:52). The flow rate and column temperature were maintained at 1.0 mL/min and 35°C, respectively. The detector was set at 430 nm for acquiring chromatograms, and the injection volume was 10 μL. The standards of bisdemethoxycurcumin, desmethoxycurcumin and curcumin were obtained from the National Institute for the Control of Pharmaceutical and Biological Products.

### 2.3 Animals

Animal experiments were approved by the Institutional Animal Care and Use Committee and the local experimental Ethics Committee (Hubei University of Chinese Medicine, 2017, Laboratory Animal Certificate no. SYXK2017‐0067). Male Sprague–Dawley rats (180–220 g, 50–70‐day‐old) and Kunming mice (18–22 g, 8‐week‐old) were obtained from the Hubei Provincial Center for Disease Control and Prevention (SCXK 2015–0018; Wuhan, China) and maintained in temperature‐controlled colony rooms (24 ± 2°C, 55 ± 15% humidity) on a 12‐h day/night cycle. Food and water were continuously available.

### 2.4 Experimental groups and copulatory behaviour testing

The male mice were randomized into six groups of eight animals each: vehicle control, hydrocortisone control, JKSQP (1.3 g/kg), three different doses of CLE (50 mg/kg, 150 mg/kg and 450 mg/kg). Besides the vehicle control group, received only normal saline, each group was intraperitoneally injected 25 mg/kg hydrocortisone for 7 days.^19^ Hydrocortisone Control Group: Animals were intraperitoneally administered 25 mg/kg hydrocortisone for 7 days starting from the 4th day of administration. JKSQP and CDL Group: Animals underwent the same treatment regimen as the Hydrocortisone Control Group but were orally administered with JKSQP or CDL for 30 days, beginning 3 days prior to hydrocortisone injection. The Vehicle Control Group received treatment with normal saline. Female mice were brought to oestrous by intragastric administration of estradiol valerate (0.4 mg/kg) 48 h and 6 h before pairing.

The copulatory behaviour tests were carried out following the modification of the procedures.^20^, ^21^ The studies were monitored in a separate room and allowed a 15 min adaptation period. Then, we introduced the female into the cage and recorded the sexual behaviour of the male for 30 min using a video camcorder. The following sexual behaviour parameters were recorded: mount latency (ML), the interval from the introduction of the female to the first mount by the male; intromission latency (IL), the interval from the introduction to the first intromission; mount frequency (MF), the number of mounts without intromission between the introduction and the ejaculation; intromission frequency (IF), the number of intromissions between the introduction and the ejaculation.

The male mice were sacrificed 24 h on the 31st day. Under ethyl ether anaesthesia, blood samples were collected from the orbital plexus. Then testis and epididymis were removed after mice were euthanized by cervical dislocation, and their organs were weighed to calculate organ coefficient (organ to body weight ratio). The serum testosterone concentrations were detected using the Microplate Enzyme Immunoassay kits, as described in the manufacturer's test procedure.

### 2.5 Histopathological study

Specimens from the testes were rapidly fixed in 10% neutral buffered formalin. The fixed sample was afterwards processed through the conventional paraffin embedding techniques and stained with haematoxylin and eosin (HE) for light microscopic examination.

### 2.6 Isolation, purification and culture of rat Leydig cell

Leydig cells were isolated from Sprague Dawley rats and cultured as previously described,^22^, ^23^ with some modifications. Briefly, the decapsulated rat testes were placed into PBS solution containing 0.05% collagenase I at 37°C for 30 min. The digestion was stopped by DMEM‐F12 culture medium containing 9% fetal bovine serum, 1% horse serum, 1% 0.5 mM sodium pyruvate and 1% penicillin–streptomycin. The digested testes were afterwards filtered through a 70‐μm nylon mesh and the Leydig cells were sequentially purified by 5%, 30%, 58% and 70% Percoll gradient separation. After centrifugation, the Leydig cells were located between Percoll gradient 70% and 58% (in the second layer from the bottom). For further experiments, the isolated Leydig cells were incubated in the DMEM‐F12 culture medium at 37°C in a 5% CO_2_ incubator.

Cell viability determined by the trypan blue test was more than 90%. The purity of Leydig cells was determined to be >90% by 3β‐hydroxysteroid dehydrogenase (3β‐HSD) histochemical staining.^24^ Dyeing reagents were the following: staining solution A (1 mL DMSO with 10 mg DHEA and NBT), staining solution B (1 mL PBS with 10 mg β‐NAD) and solution C (1 mL PBS with 1 mg niacinamide). Solutions A, B, C, and PBS were mixed in a ratio of 1:10:10:79. Cell smears were prepared and stained for 1 h. The positive cells were stained a dark blue and counted under an inverted microscope (Olympus, Tokyo, Japan).

### 2.7 Cell viability assay

Purified Leydig cells (5 × 10^3^cells/well) were cultured in 96‐well plates at 37°C with 5% CO_2_ for 48 h. The cells were then cultured in the serum‐free medium containing different doses of CLE (0.2, 1, 5, 25 μg/mL), curcumin, or 1 IU/mL human chorionic gonadotrophin (hCG) as a positive control for 24 h. The cell viability assay was evaluated using the MTT proliferation assay with three replicates.^25^ The absorbance was measured at 570 nm by a microplate reader (Synergy HT).

### 2.8 Measurement of testosterone production

We detected the testosterone from the cell supernatant, and the primary Leydig cells were plated in 24‐well plates for 24 h at a density of 5 × 10^4^ cells/well. Changing media into the serum‐free medium in the absence or presence of different doses of CLE or curcumin for 24 h. The hCG was cultured for 6 h as a positive control. According to the manufacturer's instructions, testosterone secreted into the culture medium was measured using ELISA kits.

### 2.9 Quantitative RT‐PCR

Leydig cells underwent treatment with CLE (0.2, 1, 5, 25 μg/mL) for 24 h. According to the manufactured instructions, total RNA was isolated from the testes by Trizol reagent (Invitrogen, Carlsbad, CA, USA). An ultra microspectrophotometer (Thermo Fisher Scientific, USA) was used to assess the yield and purity at 260 and 280 nm. 2 μg of total RNA was reversely transcribed into the first‐strand cDNA using a FastQuant RT kit according to the manufacturer's protocol. The primers for each gene were designed and composed by Sangon Biotech (Shanghai), as listed in Table 1. Each PCR reaction was performed with 0.5 μL of cDNA and 0.6 μL of each primer (10 μM) in a final volume of 20 μL using SuperReal PreMix Plus kits. The PCR amplification was achieved on the LightCycler 480 Real‐Time PCR system (Roche, Basel, Switzerland) as follows: 95°C for 15 min; 45 cycles of 95°C for 10 s, 60°C for 40 s and S72°C for 32 s; 72°C for 5 min; and a 4°C for hold. *Gapdh* was used as an internal control, and samples were prepared in triplicate with three independent repeats. The expression relative to control was calculated using the 2^−ΔΔ^Cq method.^26^

**TABLE 1:** The primer sequence.

Accession number	Primer	Sequence (5′–3′)	Ct (mean)
NM_002046	Gapdh	Forward: GGCTCTCTGCTCCTCCCTGT Reverce: CGTTCACACCGACCTTCACC	18.34
NM_000349	Star	Forward: GGAACCCAAATGTCAAGGAAATCA Reverce: CAGGCATCTCCCCAAAGTGTG	27.45
NM_000781	Cyp11a1	Forward: GTCCAGTTGGTCCCACTCCTC Reverce: AAGCACCAGGTCGTTCACAATATAC	32.27
NM_000862	Hsd3b1	Forward: GTACATTTATGGGGAGAGAAGTCC Reverce: CCAGGCCACATTGCCTACATA	32.52
NM_000413	Hsd17b1	Forward: GGTGGTGCTGCTGTAGAAGAT Reverce: TGTTTCAACCCCAATGACTAAG	31.75
NM_000102	Cyp17a1	Forward: GCTCCGAAGGGCAAGTAA Reverce: TCCGAGAAGTGCTGCGTAT	35.11
NM_004959	Nr5a1	Forward: TCTCTAACCGCACCATCAAG Reverce: TCGACAATGGAGATAAAGGTC	34.78

*Note*: Nuclear receptor subfamily 5 group A member 1 (Nr5a1), steroidogenic acute regulatory protein (Star), cholesterol side‐chain cleavage enzyme (Cyp11a1), 17‐alpha‐hydroxylase/17, 20‐lyase (Cyp17a1), 3β−/17β‐hydroxysteroid dehydrogenase (Hsd3b1/Hsd17b1).

### 2.10 Western blotting analysis

The effect of various doses of CLE (treatment for 24 h) on NR5A1, CREB, HSD3B1, and StAR protein expressions was detected in rat Leydig cells homogenized with RIPA lysis buffer. Vehicle‐treated cells represented the control group, and 3.0 IU/mL hCG was positive. 10 μM of cAMP−protein kinaseA (PKA) inhibitor H89 or 10 μM of StAR inhibitor melatonin were also used where appropriate. The total protein content was quantified with BCA protein assays. The equal amount of protein (20 μg per lane) was separated by 12% SDS‐polyacrylamide gel electrophoresis and electroblotted to polyvinylidene fluoride (PVDF) membranes. The membranes were incubated in blocking buffer (Tris‐buffered saline with 5% bovine serum albumin) at 4°C overnight. Then they were probed with rabbit polyclonal anti‐CREB, anti‐NR5A1, anti‐HSD3B1, anti‐StAR, and anti‐GAPDH at 4°C overnight, all diluted 1:1000 in Tris‐buffered saline. Horseradish peroxidase (HRP)–linked goat anti‐rabbit IgG (1:2000) was then probed the primary antibodies at room temperature for 1 h. The membranes were then visualized using Pierce ECL Western Blotting Substrate and scanned using a gel imaging system (FCQ, ProteinSimple, USA). Finally, the developed blots were subjected to densitometry using the GAPDH as the internal control.

### 2.11 Statistical analysis

Results were statistically analysed by one‐way analysis of variance (ANOVA) followed by Dunnett's *t*‐test using GraphPad Prism 8. Data were presented as means plus or minus the standard error of the mean (SEM). The minimum level of significance was set at *p* < 0.05. The intensity of western blot bands was quantified using Image J software.

## 3 RESULTS

### 3.1 Content analysis

**FIGURE 1:**
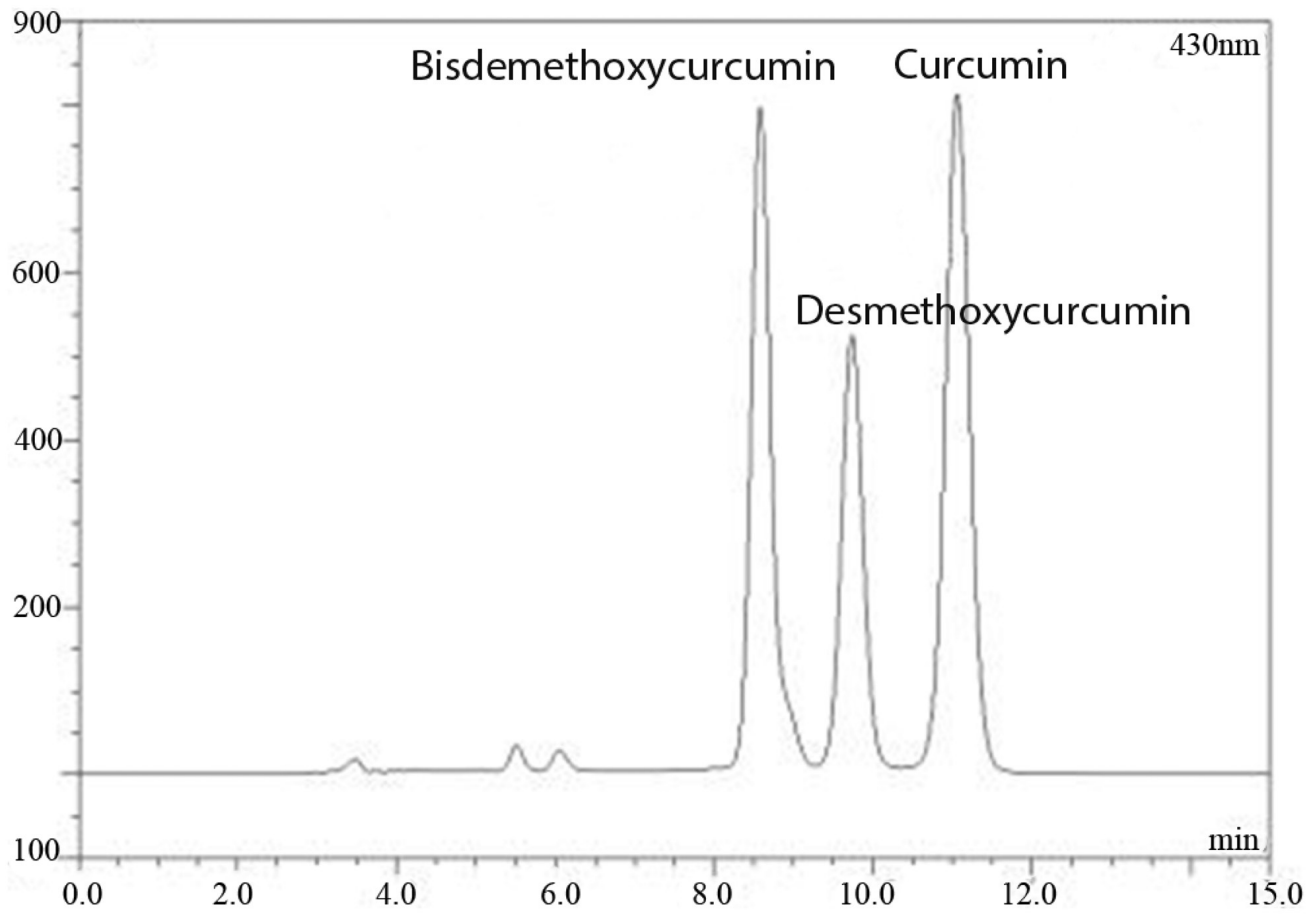
The HPLC chromatogram of *C. longa* extract.

RP‐HPLC was applied for examining curcumin content. The peaks of bisdemethoxycurcumin, desmethoxycurcumin, and curcumin were marked in Figure 1, and the curcumin content was about 12.5% of total CLE.

### 3.2 Effect of CLE on sexual behaviour

**FIGURE 2:**
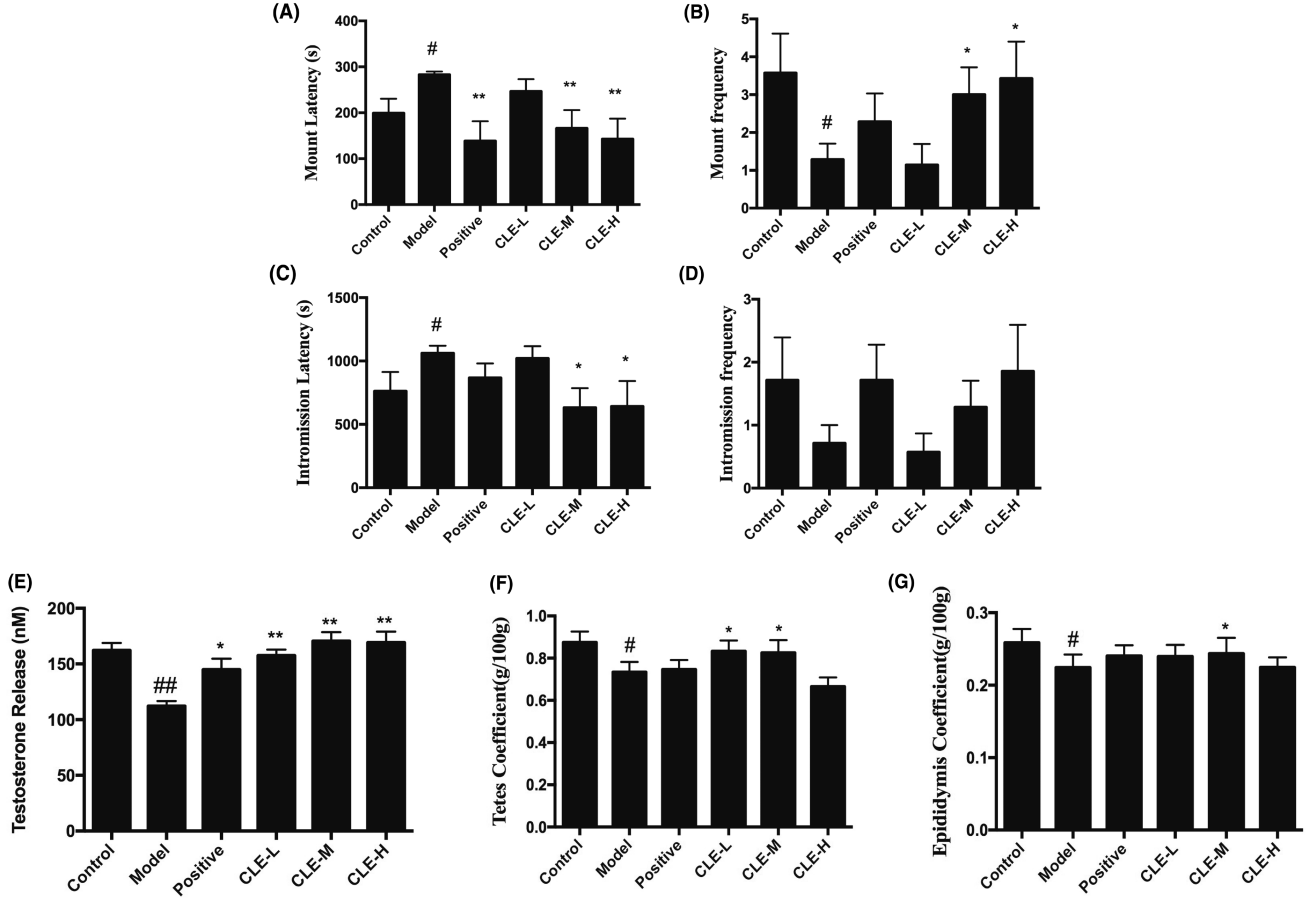
Effect of *C. longa* extract on hydrocortisone treated male mice, (A–D) Effect of *C. longa* extract on the mount latency, mount frequency, intromission latency, and intromission frequency of hydrocortisone treated male mice. (E) Effect of *C. longa* extract on the testosterone concentration of hydrocortisone treated male mice. (F, G) Effect of *C. longa* extract on organ coefficient of hydrocortisone treated male mice. #*p* < 0.05, ##*p* < 0.01 respect to control; *p < 0.05, **p < 0.01 respect to model. Control, vehicle control group; Model, hydrocortisone control group; Positive, Jinkui Shenqi Pill group; Low, low dose of *C. longa* extract group; Medium, medium dose of C. longa extract group; High, high dose of *C. longa* extract group.

When the mice were treated with hydrocortisone, they showed reduced activity, slowed reaction, dropped appetite, also the male sexual behaviour exhibited a decrease in MF and IF and a significant increase in ML and IL (*p* < 0.05) when compared with the control (Figure 2A–D). The administration of medium and high doses of the CLE resulted in a significant increase in the sexual vigour of MF and IF (*p* < 0.05) compared with the model animal group; however, the ML and IL statistically decreased significantly (*p* < 0.05 or *p* < 0.01). Besides, the JKSQP group exhibited a similar result to the medium and high doses of the CLE group.

### 3.3 Effect of CLE on serum hormone concentration

The administration of hydrocortisone significantly decreased serum testosterone levels (*p* < 0.01) compared to the control mice. However, CLE (*p* < 0.01) or JKSQP (*p* < 0.05) treated together led to a significant increase in serum testosterone concentration (Figure 2E).

### 3.4 Effect of CLE on organ coefficient

As shown in Figure 2F,G treatment with hydrocortisone significantly reduced the organ weight of testes and epididymis compared to the control mice (*p* < 0.05). After CLE were administrated together, the organ weight of the testes and epididymis significantly increased (*p* < 0.05).

### 3.5 Effect of CLE on histological changes in testicular tissue

**FIGURE 3:**
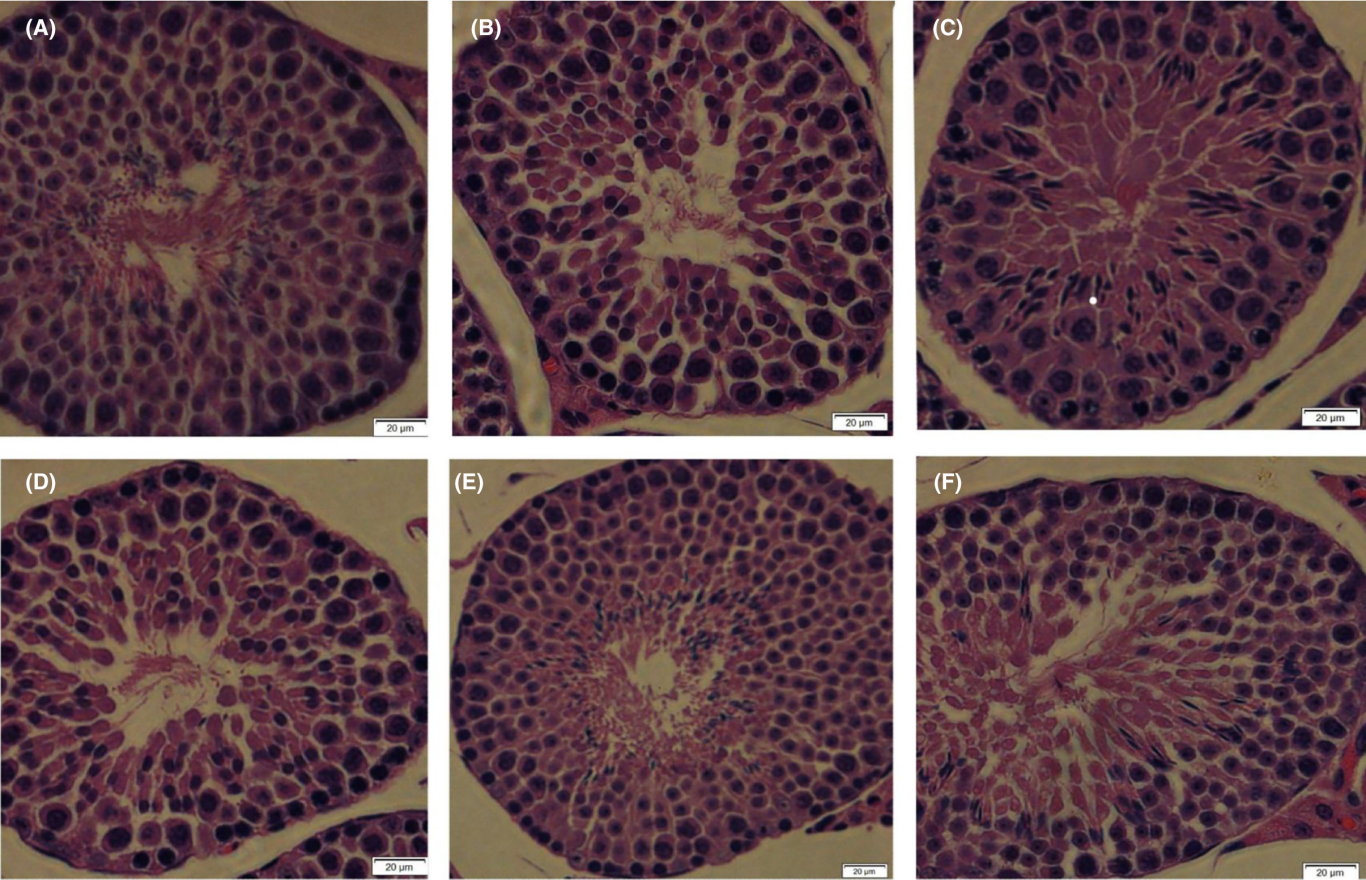
HE staining of testicular tissue in mice (400×, *n* = 8). (A) Control, (B) Model, (C) Positive, (D) *C. longa* extract‐50 mg/kg, (E) *C. longa* extract‐150 mg/kg, (F) *C. longa* extract‐ 450 mg/kg.

Testicular tissue of the control mice had a typical architecture that consisted of uniform, well organized spermatogenic epithelium and normal interstitial connective tissue in the seminiferous tubules (Figure 3). Testicular section of mice that received hydrocortisone alone showed disruption of the seminiferous epithelium's regular cellular organization, including detached and disorder of the spermatogenic cells, decreased spermatozoon. Conversely, mice that received hydrocortisone plus CLE showed a marked improvement of spermatogenesis. There were comparatively well‐organized spermatogenic layers and a moderate number of sperms in the middle‐dose and high‐dose CLE groups.

### 3.6 Leydig cell viability

**FIGURE 4:**
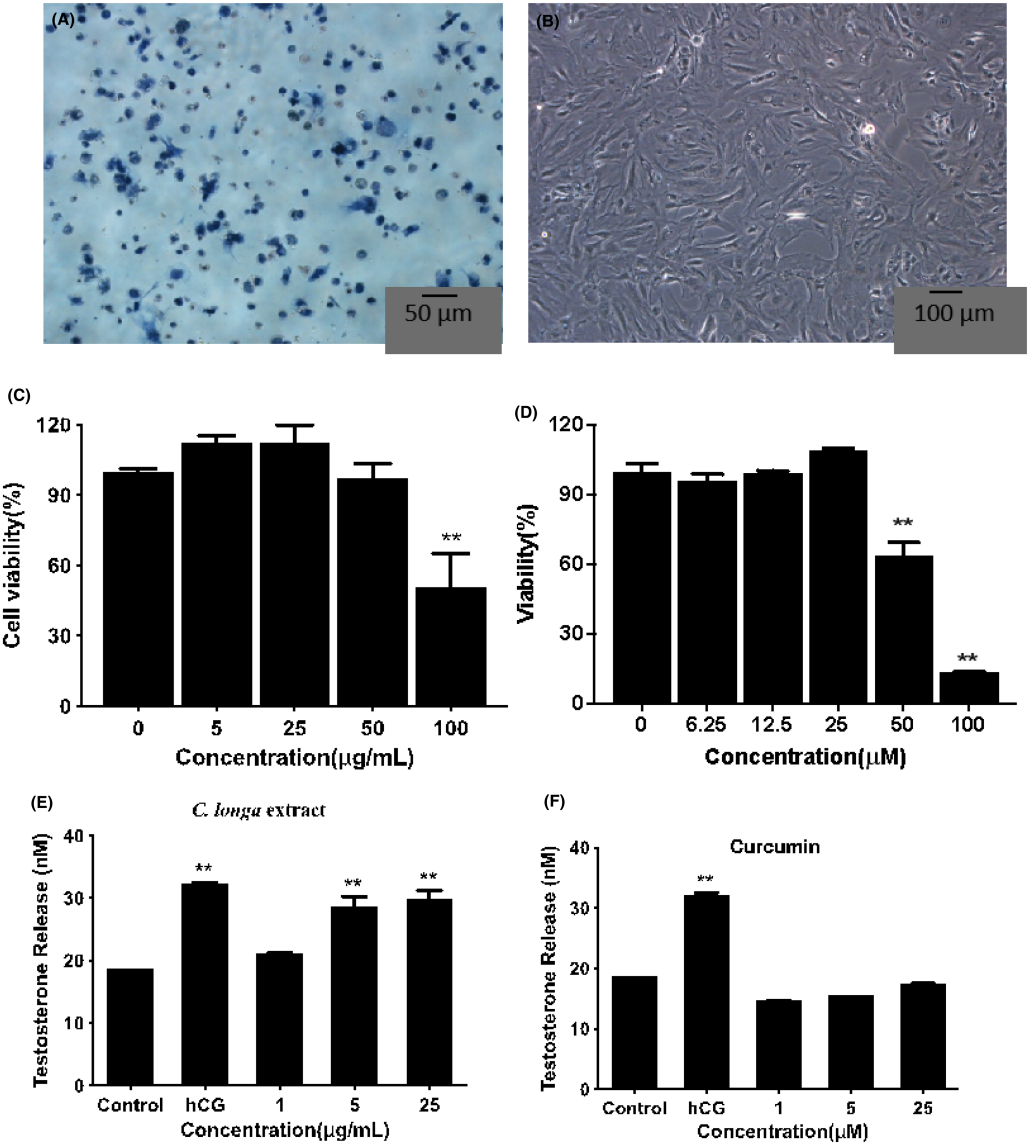
Effects of *C. longa* extract and curcumin on Leydig cells (A) 3β‐HSD staining of purified rat Leydig cells. The positive cells were stained in dark blue colour. (B) Cell morphology of rat Leydig cells. (C)The viability of Leydig cells after *C. longa* extract treatment. (D) The viability of leydig cells after curcumin treatment. (E, F) Effects of *C. longa* extract and curcumin on testosterone secretion in Leydig cells. *p < 0.05, **p < 0.01 respect to control.

Figure 4A,B showed that Leydig cells were successfully isolated from testes according to the 3β‐HSD staining test. The MTT results (Figure 4C) indicated that 100 μg/mL CLE significantly reduced the cell viability. Still, there were no apparent differences at lower concentrations (≤50 μg/mL), and the cell number was more than 90% with ≤25 μg/mL CLE. Besides, curcumin below ≤25 μM had no significant differences from the control (Figure 4D). Therefore, the subsequent experiments were performed with 25 μg/mL and lower concentrations of CLE.

### 3.7 Testosterone production

As shown in Figure 4E,F, exposure to 5 μg/mL and 25 μg/mL CLE resulted in a significant increase of testosterone secretion in rat Leydig cells, and it showed a similar result with hCG (**p < 0.01). 1 μg/mL CLE showed no apparent differences with the control group. However, curcumin had no significant effect on steroidogenesis ranging from 1 μM to 25 μM.

### 3.8 Changes in mRNA amount of transcription factor *Nr5a1* and steroidogenic enzymes

**FIGURE 5:**
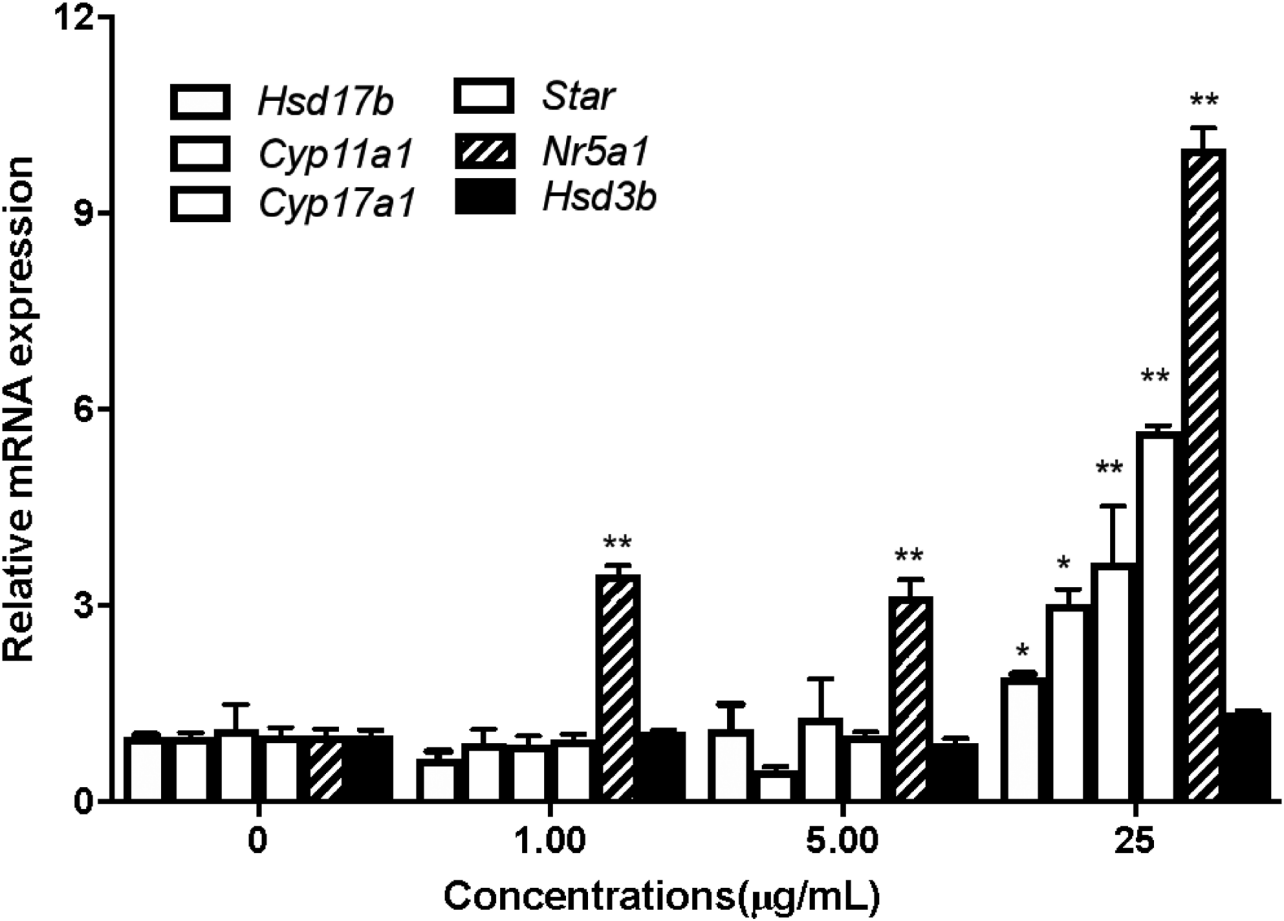
Effects of *C. longa* extract on the mRNA expression levels of *Nr5a1* and steroidogenic enzymes in Leydig cells. *p < 0.05, **p < 0.01 respect to control.

The mRNA amount of transcription factor Nr5a1 and steroidogenic enzymes (Star, Cyp11a1, Cyp17a1, Hsd3b1 and Hsd17b1) in Leydig cells were analysed using RT‐PCR analysis. Compared to the control group, the amount of mRNA of Nr5a1 showed a significant increase in the 1, 5, 25 μg/mL CLE treated group (*p* < 0.01 or *p* < 0.05). Meanwhile, the steroidogenic enzymes (Star, Cyp11a1, Cyp17a1, Hsd3b1 and Hsd17b1) also increased significantly in the 25 μg/mL CLE treated group (*p* < 0.01), as shown in Figure 5. These demonstrated that CLE could promote testosterone synthesis via up‐regulating the transcription factor and activating its downstream steroidogenic genes.

### 3.9 Protein expression levels of transcription factor NR5A1 and steroidogenic enzymes

**FIGURE 6:**
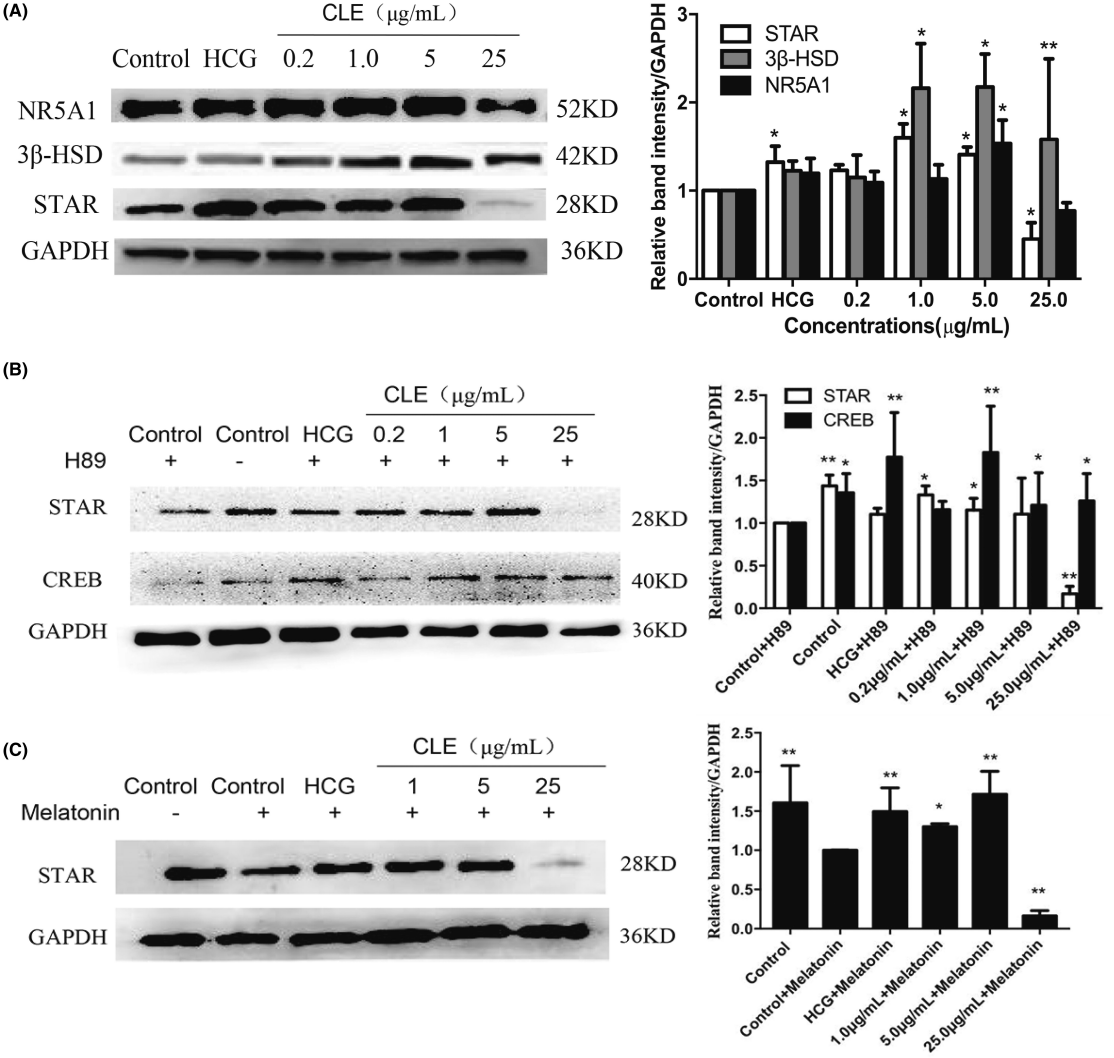
Effect of *C. longa* extract (CLE) on the protein expression in Leydig cells. (A) Effect of *C. longa* extract (CLE) on the protein expression of NR5A1, StAR, and HSD3B1 in Leydig cells. *p < 0.05, **p < 0.01 respect to control. (B, C) Effect of *C. longa* extract (CLE) on the protein expression of StAR following the addition of H89 or melatonin in Leydig cells. *p < 0.05, **p < 0.01 respect to control+Inhibitor.

We next detected the protein expression of transcription factors (NR5A1 and CREB) and steroidogenic enzymes (HSD3B1 and StAR) in Leydig cells by western blotting. The immunoblot analysis of proteins extracted from Leydig cells treated with different concentrations of CLE (0.2, 1, 5 and 25 μg/mL) demonstrated that CLE significantly promoted protein expression of HSD3B1 range from 1 μg/mL to 25 μg/mL (*p* < 0.05 or *p* < 0.01). StAR and NR5A1 also increased at 1 g/mL or 5 g/mL, compared with the control group (*p* < 0.05) (Figure 6A). However, 25 μg/mL CLE showed feedback inhibition on StAR protein expression.

H89 is a selective and potent inhibitor of PKA via competitively antagonizing the ATP site.^27^ Melatonin has an anti‐gonadal effect and is a potent androgen synthesis inhibitor in vitro.^28^ Figure 6B showed that 1‐h pretreatment with H89 resulted in a significant decrease in StAR and CREB expression than the control group (*p* < 0.05 or *p* < 0.01). However, treatment with CLE restored StAR level at the concentration of 0.2 and 1 μg/mL (*p* < 0.05), and restored CREB level range from 1 μg/mL to 25 μg/mL (*p* < 0.05 or *p* < 0.01). Similarly, the addition of melatonin to Leydig cells prevented StAR protein expression (*p* < 0.01), and 1 μg/mL or 5 μg/mL CLE could recover StAR protein expression (Figure 6C).

## 4 DISCUSSION

Turmeric, one of the major spices, is commonly used for health care, food preservation, and as a yellow dye for textiles. Its constituents include three curcuminoids (curcumin, desmethoxycurcumin, bisdemethoxycurcumin), volatile oils, sugars, proteins, and resins. Curcumin, the primary constituent of turmeric is responsible for its vibrant yellow colour and biological activity. However, its application is limited due to instability, low water solubility and poor oral bioavailability.^29^ Curcumin‐C3 has been reported to exhibit better therapeutic properties due to the synergistic effect of three curcuminoids.^30^ Additionally, a study indicated that curcumin had no impact on basal steroidogenesis in MA‐10 cells.^31^ The extract used in this research combines three curcuminoids, consisting of about 12.5% curcumin, 11.2% bisdemethoxycurcumin, and 6.8% desmethoxycurcumin. Our data also demonstrated that only the CLE could increase basal steroidogenesis in primary rat Leydig cells, while curcumin alone showed no significant effect on testosterone production.

This study investigated the efficacy of CLE on Kidney‐Yang deficiency syndrome induced by a high dose of subcutaneous hydrocortisone, which decreases steroid hormone from the adrenal cortex. Mice in the hydrocortisone group showed reduced activity, slowed reactions, decreased appetite, diminished sexual behaviour, including changes in reproductive organ indexes, testosterone concentration and increased pathological testicular damage. After CLE treatment, signs of exhaustion, mating ability with impotence, contraction of reproductive organs, testosterone levels and pathological testicular status significantly improved. These findings suggest that CLE might be a potential treatment for Kidney‐Yang deficiency syndrome, consistent with previous reports indicating that turmeric can increase sexual function and sex hormone levels.^11^, ^12^

Testosterone is synthesized from cholesterol in a multi‐step enzymatic process in response to pituitary hormones. StAR appears to be the rate‐determining step, transferring cholesterol from cellular stores to the inner mitochondrial membrane, where it is metabolized by CYP11A to pregnenolone.^32^ Pregnenolone is further converted to testosterone by other steroidogenic enzymes, such as HSD3B1, CYP17 and HSD17B1.^33^ Transcription factors like NR5A1 and pCREB regulate the expression of CYP11, HSD3B1, and StAR. Our data showed that curcuminoids stimulated testosterone production via the cAMP/PKA signalling pathway, upregulating transcription factors (NR5A1 and CREB), and steroidogenic enzymes (StAR, CYP11A1, CYP17A1, HSD3B1 and HSD17B1). However, CLE at a concentration of 25 μM showed some inhibitory effects on steroidogenic enzymes and exhibited cytotoxicity at a concentration of 50 μM. This result is consistent with previous reports indicating that in addition to the feedback inhibition of sex steroids on the HPA to suppress gonadotropin secretion, steroid hormones could induce feedback inhibition of StAR protein expression in Leydig cells.^34^

The variation in RNA and protein expression is a complex, co‐dependent process. However, except for Nr5a, the mRNA expression of steroidogenic enzymes increased only at a concentration of 25 μg/mL. There were also differences in expression variation at the RNA, protein and testosterone levels. These differences might be related to temporal elements of gene regulation, including lag times between transcription and translation and the different half‐lives of mRNA and protein molecules.^35^ Further studies are recommended to investigate these mechanisms in more detail.

H89 is a selective cAMP‐PKA inhibitor, and melatonin is a potent inhibitor of androgen synthesis through specific binding sites blocking StAR protein expression without altering the activity of P450 enzymes and cAMP.^36^, ^37^ We used H89 and melatonin to study the involvement of cAMP/PKA signalling pathways. Our data demonstrated that the CLE could markedly diminish H89‐suppressed StAR and CREB expression and melatonin‐suppressed StAR expression. This result is supported by previous evidence showing that H89 abolished the effect of curcumin treatment to increase phosphorylation of LKB‐1 and CREB. Thus, it appears that CLE stimulated testosterone production via the cAMP/PKA signalling pathway, unaffected by the PKA and StAR inhibitors.

## 5 CONCLUSION

CLE could reverse hydrocortisone‐induced Kidney‐Yang deficiency syndrome by improving sexual behaviour, reproductive organ weight, testosterone levels, and reducing pathological damage. The in vitro study further indicated that CLE stimulated testosterone production by upregulating mRNA or protein expression of transcription factor (NR5A1 and CREB) and steroidogenic enzymes (StAR, CYP11A1, CYP17A1, HSD3B1 and HSD17B1). CLE could diminish H89‐suppressed StAR and CREB expression, and melatonin‐suppressed StAR expression. The data from this study suggest that CLE could reverse Kidney‐Yang deficiency symptoms and stimulate testosterone production by upregulating the cAMP/PKA signalling pathway, indicating its potential as a nutraceutical choice against testosterone deficiency diseases.

## AUTHOR CONTRIBUTIONS

**Yisheng Zhang:** Writing – original draft (lead). **Liu Yang:** Data curation (equal). **Shan Xue:** Data curation (equal); writing – original draft (equal). **Yichang Zhang:** Data curation (equal). **Zihan Li:** Writing – original draft (equal). **Min Zhang:** Methodology (equal). **Guoyin Kai:** Writing – review and editing (equal). **Juan Li:** Methodology (lead); writing – original draft (equal).

## CONFLICT OF INTEREST STATEMENT

The authors declare that there are no conflicts of interest.

## Data Availability

The datasets used and analyzed during the current study are available from the corresponding author upon reasonable request.
